# Comparison of two commercial tests (Immy vs. Dynamiker) for cryptococcal capsular antigen

**DOI:** 10.1590/0037-8682-0307-2021

**Published:** 2021-09-06

**Authors:** María Clara Noguera, Patricia Escandón, Javier Rodríguez, Alexander Parody, Leidy Camargo

**Affiliations:** 1 Universidad Metropolitana, Grupo Caribe de Enfermedades Infecciosas, Barranquilla, Atlántico, Colombia.; 2 Instituto Nacional de Salud, Grupo Microbiología, Bogotá, Colombia.; 3 Universidad Metropolitana, Semillero SIPBAC Programa de Bacteriología, Barranquilla, Atlántico, Colombia.

**Keywords:** Lateral flow assay, CrAg-LFA, IMMY, Dynamiker, Point-of-Care test, Diagnostic performance

## Abstract

**INTRODUCTION:**

Lateral flow assay is an advanced method useful in the early diagnosis of cryptococcal meningitis. We aimed to compare two commercial tests for cryptococcal capsular antigen in the sera of asymptomatic patients with human immunodeficiency virus in Barranquilla, Colombia.

**METHODS:**

Thawed (n=162) previously collected serums (2016-2019) were processed using IMMY and Dynamiker cryptococcal antigen lateral flow assay.

**RESULTS:**

Compared to IMMY’s results, Dynamiker’s sensitivity, specificity, positive predictive value, negative predictive value, and kappa index were 100%, 89.9%, 48.3%, 100.0%, and 0.61, respectively.

**CONCLUSIONS:**

The Dynamiker test had excellent sensitivity, acceptable specificity, and a low detection threshold for cryptococcal antigen in the tested samples.

Globally, *Cryptococcus neoformans* var. *grubii*, is the main pathogenic species responsible for meningitis in patients with acquired immune deficiency syndrome (AIDS). Moreover, cryptococcosis is an important cause of morbidity and mortality, especially in middle- and low-income countries, and is estimated to cause 15% of AIDS-related deaths worldwide^1^ ^,^ ^2^. 

Over time, standard diagnostic methods for cryptococcal meningitis have progressed from India ink visualization and culture to the detection of circulating antigens through latex agglutination tests, Enzyme-Linked ImmunoSorbent Assay (ELISA), and more recently, cryptococcal antigen lateral flow assay (CrAg LFA). CrAg-LFA is a point-of-care (POC) test that uses a dipstick with monoclonal antibodies that can detect the capsular antigen in the four serotypes (A, B, C, D) of the pathogenic *Cryptococcus neoformans* species complex and the *C. gattii* species complex. CrAg-LFA has been validated in serum and cerebrospinal fluid (CSF) with an analytical performance close to 100%; the greatest benefit of this test is its ability to detect minimal amounts of circulating antigen during the prodromal phase (average 22 days prior to symptoms) allowing timely treatment^2^ ^-^ ^5^. 

In countries with a 3% or more prevalence of cryptococcosis, the World Health Organization (WHO) recommends using this screening immunochromatographic test for the early diagnosis of cryptococcal disease in adults with advanced human immunodeficiency virus (HIV) disease who are not undertaking antiretroviral therapy (ART) and have a CD4+ level of <100 cells/µL (eventually, CD4 <200 cells/µL). If the test result is positive, secondary prophylaxis with fluconazole (FLU) is indicated (200 mg daily); Thereafter, ART is considered^4^. 

This test is rapid, simple, cost-effective, and does not require laboratory infrastructure; these attributes can improve the diagnostic capacity in regions with a high prevalence of cryptococcosis. Further, as CrAg-LFA is a POC test, trained workers can perform it either in a health care facility or at the patient’s bedside and obtain results after 10 minutes^2^ ^,^ ^3^. 

Since 2018, several manufacturers from the United States, France, and China have made CrAg-LFA kits available with a similar principle of a dipstick sandwich immunochromatographic assay that takes place on nitrocellulose test strips; all procedures are applicable to serum and CSF samples, and some manufacturers provide kits that are also applicable to plasma and whole blood (venous and finger stick) samples^3^ ^,^ ^6^. 

Regarding antigenemia for *Cryptococcus*, a retrospective comparative study in Colombia by Escandón et al. with 421 serum samples from HIV patients, showed a higher sensitivity of CrAg-LFA when compared with CrAg Latex^7^. In Colombia, meningeal cryptococcosis is the second most frequent opportunistic infection of the central nervous system (CNS) in the HIV population^8^. In many cases, patients remain unaware of their condition; this limits the provision of timely care. The incidence of HIV in Colombia has increased since 2008 and Barranquilla (capital city of the Department of Atlántico), was ranked fifth in HIV/AIDS incidence (2018) above the national average (27.3×10^5^ inhabitants) and was the city on the Colombian Caribbean coast with the second highest number of reported cases^9^. Additionally, the prevalence of cryptococcosis in this department between 2015-2017 (5.08×10^6^ inhabitants) was almost five times that in a previous report (1.04×10^6^ inhabitants) that spanned 17 years, between 1997-2014^8^. 

The aim of this study was to establish the diagnostic performance of two CrAg-LFA POC tests in the sera of asymptomatic HIV patients in Barranquilla, Colombia.

This study was carried out with 162 cryopreserved (-20°C) serum samples from asymptomatic HIV patients with CD4+ ≤120 cells/μL collected from July 2016 to May 2019 for a previous study during which none of the patients developed cryptococcosis^10^. The specimens were blindly tested by a trained laboratory technologist following the manufacturer’s instructions for two LFA tests: IMMY Cryptococcal Antigen LFA (Immuno-Mycologics, Norman, OK) (LFA-I) and Dynamiker Cryptococcal Antigen LFA (Tianjin Co., Ltd.) (LFA-D)^11^ ^,^ ^12^. To compare the diagnostic performance of both immunoassays, LFA-I was considered the reference test given the many multisite validation studies performed for it, as well as its international endorsements from the United States Food and Drug Administration (FDA), and its European Conformity (CE) marking in Europe^3^; LFA-D was considered the diagnostic test.

Statistical analysis was performed using the Statgraphics Centurion XVI statistical software (Statgraphics Technologies, Inc., The Plains, Va, USA). The descriptive results were based on absolute and relative frequency tables. The level of convergence between the two tests (IMMY and Dynamiker) was analyzed by calculating the sensitivity, specificity, positive predictive value (PPV), negative predictive value (NPV), and kappa concordance index using a confidence level of 95%.

Of the 162 serum samples that were processed using the LFA-I assay, 14 tested positive and 148 tested negative. Furthermore, of the 162 samples that were processed with LFA-D, 29 tested positive and 133 tested negative.

Regarding the diagnostic performance and the agreement between both tests, the sensitivity of LFA-D was 100%, specificity was 89.9%, and accuracy was 90.7%. As presented in Table 1, 100% (14/14) of the positive cases detected by the reference test (LFA-I) were also identified as positive by LFA-D and 89.9% (133/148) of the cases identified as negative by LFA-I) were identified as negative by LFA-D. However, 9.25% (15/162) samples with a positive result by LFA-D were tested negative by LFA-I; we suppose these were false positives. Furthermore, the PPV of LFA-D was 48.3% (14/29) while the NPV was 100% (133/133). The calculated Cohen's kappa index showed good agreement with a result of 0.61.

**TABLE 1:** Cryptococcal antigen detection by LFA for 162 serum samples - Barranquilla, Colombia (2019).

	LFA-I Positive	LFA-I Negative	Total
**LFA-D Positive**	14	15	29
**LFA-D Negative**	0	133	133
**Total**	14	148	162

**LFA-I:** Lateral Flow Assay- IMMY; **LFA-D:** Lateral Flow Assay- Dynamiker.

Meningeal cryptococcosis is one of the most important causes of meningitis in adults, especially in regions with high rates of HIV infection^2^ ^,^ ^3^. The detection of cryptococcal capsular polysaccharide antigen with qualitative/semiquantitative results is the most sensitive diagnostic tool for identifying cryptococcosis weeks to months prior to the onset of meningitis symptoms^2^ ^,^ ^4^. Because of its many advantages, early diagnosis through CrAg-LFA is increasing the use of this assay, especially in resource-limited laboratory settings. Additionally, this test is recommended by the WHO as the preferred method for the diagnosis of cryptococcal disease since it meets the affordable, sensitive, specific, user-friendly, rapid/robust, equipment-free, and delivered criteria for diagnostic tests^2^ ^,^ ^4^. 

We compared the agreement between two CrAg-LFA tests from different manufacturers: the LFA-I test, which has multiple validation studies and recognition from the FDA as well as from the CE, and the LFA-D test. Despite validation studies underway since 2018, LFA-D has not yet been approved by any regulatory body in Europe or the United States^3^. Nevertheless, LFA-D has two practical advantages: no sample dilution is required, and each test is individually packaged to prevent degradation or contamination. Both tests have similar principles, but one difference is the limit of detection (LOD); the LOD for IMMY is 1.75 ng/mL whereas the LOD for Dynamiker is 1.25 ng/mL^11^ ^,^ ^12^. 

A poster was presented at the 28th European Congress of Clinical Microbiology and Infectious Diseases in Madrid, Spain (2018) regarding the evaluation of LFA-D in comparison with LFA-I and the Meridian latex agglutination test; the authors compared the performance of the latex agglutination test with two CrAg-LFA devices (IMMY and Dynamiker), concluding that both LFA tests performed excellently. In general, the band intensity was stronger for the Dynamiker CrAg-LFA. A second poster, presented during the International Congress of Pathology and Laboratory Medicine in Kuala Lumpur, Malaysia (2018), evaluated LFA-D compared to LFA-I; the authors concluded that both tests performed satisfactorily (100% sensitivity, 89.5% specificity) and further identified a strong agreement between both tests^13^ ^,^ ^14^. The sensitivity (100%) and specificity (89.9%) of LFA-D in our study were consistent with these previous data and with the values established by the WHO (100% and 96.8%, respectively)^4^. 

In this study, we assumed that the LFA-D test had a higher rate of identification of positive cases compared to LFA-I. This finding was also verified by the total number of positive cases identified by each test: 14 positive samples were identified by LFA-I, and 29 samples were identified as positive by LFA-D. Thus, slightly more than twice the number of positive samples were found when using LFA-D. This result could be explained by the different minimum identification thresholds or LODs that each test specifies; therefore, it is expected that the LFA-D test will identify a greater number of positive samples than LFA-I^11^ ^,^ ^12^. This difference could also explain the lack of high concordance between the tests used in this study. 

Kwizera et al. evaluated the diagnostic performance of the Dynamiker CrAg-LFA compared to the IMMY CrAg-LFA (reference standard) in serum, plasma, and CSF samples from symptomatic and asymptomatic HIV patients. Their study demonstrated acceptable sensitivity (96%) for the Dynamiker CrAg-LFA tested in serum samples from suspected asymptomatic patients. Their finding is comparable to our result (100%); however, the specificity (66%) reported was poorer than the specificity in our study^15^. In addition, the bands in the Dynamiker CrAg-LFA test strips in positive cases were also more intense, while the bands in the IMMY CrAg-LFA test strips were weaker; however, when the Dynamiker CrAg-LFA test strips displayed weak positive bands, the IMMY CrAg-LFA test strips were negative. We agree that the difference in the LOD between both tests could explain these differences and the high numbers of supposed false positives reported in this study^15^.

Taking our results into account, we consider it necessary and especially important to conduct future cohort studies with patients for whom cryptococcosis has been confirmed and with non-diseased patients, to compare the results for the detection of cryptococcal antigens. We conclude that, there was good concordance between both tests in our study. The Dynamiker CrAg-LFA test exhibited excellent sensitivity and acceptable specificity in addition to having a lower detection threshold for cryptococcal antigens in the tested cryopreserved serum samples.

Over the past years, early diagnosis of cryptococcosis using this POC test has dramatically improved the efforts for preventing cryptococcal meningitis. We believe that this POC test could provide a key strategy to reduce the morbidity and mortality of meningeal cryptococcosis in several regions of the world including Sub-Saharan Africa, Asia, the Pacific region, Latin America and the Caribbean, where the prevalence of cryptococcal meningitis is the highest.
